# Antitumor activity of the combination of an HSP90 inhibitor and a PI3K/mTOR dual inhibitor against cholangiocarcinoma

**DOI:** 10.18632/oncotarget.1706

**Published:** 2014-03-26

**Authors:** Ming-Huang Chen, Kun-Chun Chiang, Chi-Tung Cheng, Shih-Chiang Huang, Yeng-Yang Chen, Tsung-Wen Chen, Ta-Sen Yeh, Yi-Yin Jan, Hsi-Ming Wang, Jiang-Jie Weng, Peter Mu-Hsin Chang, Chun-Yu Liu, Chung-Pin Li, Yee Chao, Ming-Han Chen, Chi-Ying F. Huang, Chun-Nan Yeh

**Affiliations:** ^1^ Faculty of Medicine, National Yang-Ming University, Taipei, Taiwan; ^2^ Division of Hematology and Oncology, Department of Medicine, Taipei Veterans General Hospital, Taipei, Taiwan; ^3^ General Surgery Department, Chang Gung Memorial Hospital, Keelung, Taiwan; ^4^ Department of Surgery, Lin-Kou Medical Center, Chang Gung Memorial Hospital and University, Taoyuan County, Taiwan; ^5^Department of Pathology, Chang Gung Memorial Hospital and University, Taoyuan County, Taiwan; ^6^ Division of Hematology and Oncology, Department of Internal Medicine, Kaohsiung Chang Gung Memorial Hospital, Kaohsiung, Taiwan; ^7^ Division of Gastroenterology, Department of Medicine, Taipei Veterans General Hospital, Taipei, Taiwan; ^8^ Cancer Center, Taipei Veterans General Hospital, Taipei, Taiwan; ^9^ Institute of Clinical Medicine, Institute of Biopharmaceutical Sciences, and Genome Research Center, Yang-Ming University, Taipei, Taiwan

**Keywords:** NVP-AUY922, NVP-BEZ235, heat-shock protein 90, phosphatase and tensin homolog

## Abstract

The PI3K/Akt/mTOR pathway is overactivated and heat shock protein (HSP) 90 is overexpressed in common cancers. We hypothesized that targeting both pathways can kill intrahepatic cholangiocarcinoma (CCA) cells. HSP90 and PTEN protein expression was evaluated by immunohistochemical staining of samples from 78 patients with intrahepatic CCA. CCA cell lines and a thioacetamide (TAA)-induced CCA animal model were treated with NVP-AUY922 (an HSP90 inhibitor) and NVP-BEZ235 (a PI3K/mTOR inhibitor) alone or in combination.

Both HSP90 overexpression and loss of PTEN were poor prognostic factors in patients with intrahepatic CCA. The combination of the HSP90 inhibitor NVP-AUY922 and the PI3K/mTOR inhibitor NVP-BEZ235 was synergistic in inducing cell death in CCA cells. A combination of NVP-AUY922 and NVP-BEZ235 caused tumor regression in CCA rat animal model. This combination not only inhibited the PI3K/Akt/mTOR pathway but also induced ROS, which may exacerbate the vicious cycle of ER stress. Our data suggest simultaneous targeting of the PI3K/mTOR and HSP pathways for CCA treatment.

## INTRODUCTION

Intrahepatic cholangiocarcinoma (CCA) is a relatively rare, but increasing more common, hepatobiliary cancer [1–3]. CCA, the second most common liver cancer, is an aggressive cancer typically diagnosed at an advanced stage with poor prognosis [4].

The ABC-02 trial showed improved overall survival of CCA patients treated with gemcitabine plus cisplatin compared to those treated with gemcitabine alone (11.7 versus 8 months), and defined gemcitabine plus platinum as a standard treatment for advanced biliary tract cancer [5]. Several molecular-targeted therapies have been assessed in clinical trials, with median progression-free survivals of 1.8–7 months [6, 7]. However, there is no standard therapy for refractory CCA, and additional therapeutic drugs are needed urgently.

The molecular chaperone heat shock protein 90 (HSP90) plays an important role in post-translational maturation and activation of many oncogenic client proteins that increase the survival, growth, and invasive potential of cancer cells [8, 9]. HSP90 inhibitors induce ubiquitination and proteosomal degradation of numerous oncoproteins, which consequently inhibit cancer cell growth and survival [10]. We had previously demonstrated that the HSP90 inhibitor NVP-AUY922 exerted antitumor effects in CCA cells in pathways involving the phosphatidylinositol 3-kinase catalytic subunit α (PI3K)/v-Akt murine thymoma viral oncogene homolog 1 protein kinase (Akt)/mammalian target of rapamycin (mTOR) and the v-Ki-ras2 Kirsten rat sarcoma viral oncogene (KRAS)/mitogen-activated protein kinase (MAPK) pathways [11]. Although HSP90 inhibitors are considered a novel therapeutic strategy for several cancers pre-clinically [11–15], HSP90 inhibitor mono-therapy has shown only limited clinical success [16, 17] due to suboptimal inhibition of target client proteins [18]. Combination approaches may be required for effective clinical use of HSP90 inhibitors [19, 20].

The PI3K/Akt/mTOR signaling cascade is central to cell survival, apoptosis, metabolism, motility, and angiogenesis [21]. This pathway is up-regulated in CCA [22] and is a key pathway for CCA drug development [6, 7]. *PIK3CA* (phosphoinositide 3-kinase, catalytic, α-polypeptide) activating mutations are rarely found in CCA [22], suggesting that additional mechanisms are involvement. Recently, an imidazo [4, 5-c] quinoline derivative dual PI3K/mTOR inhibitor, NVP-BEZ235 was shown to inhibit CCA cell growth [23]. However, similar to HSP90 inhibitor mono-therapy, PI3K inhibitor mono-therapy has not produced significant clinical responses [21, 24].

NVP-BEZ235 targets PI3K and mTOR equally in cancer [25], and we postulated that the combination of a PI3K/mTOR inhibitor and an HSP90 inhibitor might cooperatively inhibit tumor cell proliferation and induce apoptosis. Furthermore, the HSP90 inhibitor also induces endoplasmic reticulum (ER) stress, which leads to mitochondrial damage and subsequent apoptosis [26]. This process may be fueled by oxidative stress when combined with an mTOR inhibitor [27]. Therefore, the combination of an HSP90 inhibitor and a PI3K/mTOR dual inhibitor may promote irreversible ER stress and induce cell death.

Here, we investigated the effects of targeting the PI3K/mTOR pathway (with NVP-BEZ235) and HSP90 (with NVP-AUY922) in CCA, both *in vitro* and *in vivo*.

## RESULTS

### HSP90 and PTEN expression in human CCA

**Figure 1:**
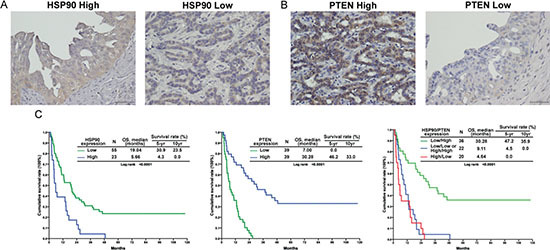
HSP90 and PTEN expression was correlated with survival in 78 patients with resectable MF-CCA **(A, B)** Immunohistochemical staining of MF-CCA tumors with different intensity scores for HSP90 and PTEN expression; **(C)** The high-HSP90 group showed significantly worse overall survival (*P* < 0.001, left); The low-PTEN group showed significantly worse overall survival (*P* < 0.001, middle); The combined high-HSP90 and low-PTEN group showed the worst overall survival (*P* < 0.001, right).

HSP90 and PTEN were diffusely expressed in the cytoplasm in human MF-CCA (Fig 1A, 1B). Two representative examples showing HSP90 and PTEN immunohistochemical staining in human CCA were graded from 0 to 3+, with 0 and 1+ indicating low expression and 2+ and 3+ indicating high expression (Figure 1A to 1B).

### Clinicopathological features and HSP90 and PTEN expression levels in patients with MF-CCA

Of the 78 specimens obtained from MF-CCA patients, 55 (70.5%) showed high HSP90 expression (2+ and 3+ positive), while 39 (50%) showed low PTEN expression. Clinicopathological features were similar between patients with low and high HSP90 and PTEN expression (Table 1 and 2).

**Table 1:** Clinicopathological features of 78 patients with high and low heat shock protein 90 (HSP90)-expressing mass-forming cholangiocarcinomas

Factor	HSP90 high (n = 55)	HSP90 low (n = 23)	*P*
Age (years)	58.3 ± 12.2	63.2 ± 10.9	0.086
Gender			0.713
Male	24 (43.6)	9 (39.1)	
Female	31 (56.4)	14 (60.9)	
Symptom			0.097
Positive	44 (80.0)	22 (95.7)	
Negative	11 (20.0)	1 (4.3)	
AST (IU/L)			0.564
≤ 34	29 (52.7)	10 (45.5)	
> 34	26 (47.3)	12 (54.5)	
ALT (U/L)			0.357
≤ 36	27 (52.9)	13 (65.0)	
> 36	24 (47.1)	7 (35.0)	
ALP (U/L)			0.491
≤ 94	15 (28.3)	8 (36.4)	
> 94	38 (71.7)	14 (63.6)	
Bilirubin (total) (mg/dL)			0.934
≤ 1.3	45 (81.8)	19 (82.6)	
> 1.3	10 (18.2)	4 (17.4)	
Albumin (g/dL)			0.226
≤ 3.5	13 (26.5)	9 (40.9)	
> 3.5	36 (73.5)	13 (59.1)	
Serum CEA (ng/mL)			0.984
≤ 5	17 (44.7)	8 (44.4)	
> 5	21 (55.3)	10 (55.6)	
Size (cm)			0.843
≤ 5	23 (43.4)	9 (40.9)	
> 5	30 (56.6)	13 (59.1)	
Lymph node			0.076
Negative	38 (69.1)	11 (47.8)	
Positive	17 (30.9)	12 (52.2)	
Differentiated			0.131
Well	1 (1.8)	1 (4.3)	
Moderate	29 (52.7)	12 (52.2)	
Poorly	25 (45.5)	8 (34.8)	
Other	0	2 (8.7)	
Post-op chemotherapy			0.254
No	21 (38.2)	12 (52.2)	
Yes	34 (61.8)	11 (47.8)	
Post-op radiotherapy			0.266
No	46 (83.6)	22 (95.7)	
Yes	9 (16.4)	1 (4.3)	

**Table 2:** Clinicopathological features of 78 patients with high and low phosphatase and tensin homolog (PTEN)-expressing mass-forming cholangiocarcinoma patients

Factor	PTEN high (n = 39)	PTEN low (n = 39)	*P*
Age (years)	61.2 ± 10.8	58.3 ± 13.0	0.294
Gender			0.492
Male	15 (38.5)	18 (46.2)	
Female	24 (61.5)	21 (53.8)	
Symptom			0.209
Positive	35 (89.7)	31 (79.5)	
Negative	4 (10.3)	8 (20.5)	
AST (IU/L)			0.570
≤ 34	19 (48.7)	21 (53.8)	
> 34	20 (51.3)	18 (46.2)	
ALT (U/L)			0.820
≤ 36	22 (56.4)	21 (53.8)	
> 36	17 (43.6)	18 (46.2)	
ALP (U/L)			0.240
≤ 94	10 (25.6)	14 (35.9)	
> 94	29 (74.4)	25 (64.1)	
Bilirubin (total) (mg/dL)			0.555
≤ 1.3	33 (84.6)	31 (79.5)	
> 1.3	6 (15.4)	8 (20.5)	
Albumin (g/dL)			0.089
≤ 3.5	16 (41.0)	9 (23.1)	
> 3.5	23 (59.0)	30 (76.9)	
Serum CEA (ng/mL)			0.063
≤ 5	14 (35.9)	17 (58.6)	
> 5	25 (64.1)	12 (41.4)	
Size (cm)			0.109
≤ 5	13 (33.3)	20 (51.3)	
> 5	26 (66.7)	19 (48.7)	
Lymph node			0.101
Negative	21 (53.8)	28 (71.8)	
Positive	18 (46.2)	11 (28.2)	
Differentiated			0.188
Well	2 (5.1)	0	
Moderate	21 (53.8)	20 (51.3)	
Poorly	14 (35.9)	19 (48.7)	
Other	2 (5.1)	0	
Post-op chemotherapy			0.819
No	16 (41.0)	17 (43.6)	
Yes	23 (59.0)	22 (56.4)	
Post-op radiotherapy			0.498
No	33 (84.6)	35 (89.7)	
Yes	6 (15.4)	4 (10.3)	

### Survival and prognostic analysis of MF-CCA patients who underwent a hepatectomy

Seventy-eight MF-CCA patients who had undergone hepatectomy were enrolled in the survival analysis study. The follow-up duration ranged from 1.4 to 94.1 (median, 13.6) months. Overall survival (OS) rates at 1, 3, and 5 years were 55.1%, 22.9%, and 14.9%, respectively (data not shown). Univariate log-rank analysis identified the following factors as negatively affecting OS: the presence of symptoms, high preoperative alkaline phosphatase and carcinoembryonic antigen levels, low albumin levels, tumor size >5 cm, positive surgical margin status, and high HSP90 and low PTEN expression (Table 3). Multivariate Cox proportional hazard analysis revealed that poor nutritional status, positive margin status, and high HSP90 and low PTEN expression independently predicted unfavorable OS in MF-CCA patients after hepatectomy (Figure 1C and Table 4).

**Table 3:** Univariate analysis of factors influencing the overall survival of 78 MF-CCA patients

Factor	Survival (months)				*P*
	Median	95% CI of median	3-year (%)	5-year (%)	
Gender					0.719
Male (n = 33)	14.70	7.89–21.50	21.2	18.2	
Female (n = 45)	10.82	5.85–15.79	24.4	15.0	
Symptoms					0.006
Negative (n = 12)	37.71	4.78–70.64	58.3	41.7	
Positive (n = 66)	10.46	5.84–15.07	16.7	11.9	
AST (IU/L)					0.197
≤ 34 (n = 39)	13.32	9.53–17.10	30.8	22.8	
> 34 (n = 38)	10.72	2.28–19.16	15.8	10.5	
ALT (IU/L)					0.625
≤ 36 (n = 40)	12.99	6.42–19.56	25.0	19.7	
> 36 (n = 31)	14.70	7.81–21.58	19.4	9.7	
ALP (IU/L)					0.009
≤ 94 (n = 23)	23.90	11.66–36.15	39.1	29.8	
> 94 (n = 52)	9.11	4.93–13.29	15.4	9.6	
Bil (total) (mg/dL)					0.581
≤ 1.3 (n = 64)	12.99	6.41–19.56	25.0	16.9	
> 1.3 (n = 14)	10.72	0.00–22.23	14.3	14.3	
Albumin (g/dL)					0.043
≤ 3.5 (n = 22)	4.70	3.12–6.29	18.2	13.6	
> 3.5 (n = 49)	19.04	13.31–24.76	24.5	15.9	
Serum CEA (ng/dL)					0.043
≤ 5 (n = 25)	18.51	2.09–34.93	40.0	27.0	
> 5 (n = 31)	10.29	4.05–16.53	6.5	6.5	
Margin					< 0.0001
Negative (n = 54)	19.43	14.97–23.89	33.3	23.8	
Positive (n = 24)	4.41	2.43–6.38	0.0	0.0	
Size					0.006
≤ 5cm (n = 32)	19.99	13.75–26.23	37.5	30.7	
> 5cm (n = 43)	9.11	1.97–16.25	14.0	7.0	
Lymph node					0.091
Negative (n = 49)	19.43	13.70–25.16	28.6	18.0	
Positive (n = 29)	10.46	0.00–22.71	13.8	13.8	
Histology					0.207
Well (n = 2)	2.73		0.0	0.0	
Moderate (n = 41)	13.84	7.45–20.24	22.0	17.1	
Poor (n = 33)	12.99	5.25–20.72	27.3	17.3	
Others* (n = 2)	4.37		0.0	0.0	
HSP90 expression					<0.0001
Low (n = 55)	19.04	11.66–26.41	30.9	23.5	
High (n = 23)	5.66	3.55–7.77	4.3	0.0	
PTEN expression					< 0.0001
Low (n = 39)	7.00	3.95–10.06	0.0	0.0	
High (n = 39)	30.28	12.30–48.26	46.2	33.0	
HSP90/PTEN expression					< 0.0001
Low/high (n = 36)	30.28	13.27–47.29	47.2	35.9	
Low/low or high/high (n = 22)	9.11	5.03–13.19	4.5	0.0	
High/low (n = 20)	4.64	1.90–7.37	0.0	0.0	

HSP90: heat shock protein 90; PTEN: phosphatase and tensin homolog; AST: aspartate aminotransferase; ALT: alanine aminotransferase; ALP: alkaline phosphatase; CEA: carcinoembryonic antigen; CA 19–9: carbohydrate antigen; IU: international unit; op: operation

* cystadenocarcinoma:1, musinous:1

**Table 4:** Cox's proportional hazards analysis

Factor	Hazard ratio (95% confidence interval)	*P*
Symptoms (positive/negative)	1.469 (0.307–7.024)	0.630
Alkaline phosphatase (U/L) (> 94/≤ 94)	1.742 (0.671–4.524)	0.254
Albumin (g/dL) (≤ 3.5/> 3.5)	2.497 (1.001–6.297)	0.049
CEA (ng/dL) (> 5/≤ 5)	1.306 (0.542–3.148)	0.552
Tumor size (cm) (> 5/≤ 5)	1.065 (0.387–2.934)	0.903
Margin (positive/negative)	2.543 (1.13–4.352)	0.043
Lymph node status (positive/negative)	0.868 (0.371–2.034)	0.745
HSP90, PTEN (low, low or high, high/low, high)	5.820 (2.138–15.844)	0.001
(high, low/low, high)	5.471 (1.930–15.507)	0.001
Post-op radiotherapy (with/without)	1.494 (0.454–4.922)	0.509

HSP90: heat shock protein 90; PTEN: phosphatase and tensin homolog; NS: not significant

### The combination of NVP-AUY922 and NVP-BEZ235 blocked proliferation and induced cytotoxicity of CCA cell lines

**Figure 2:**
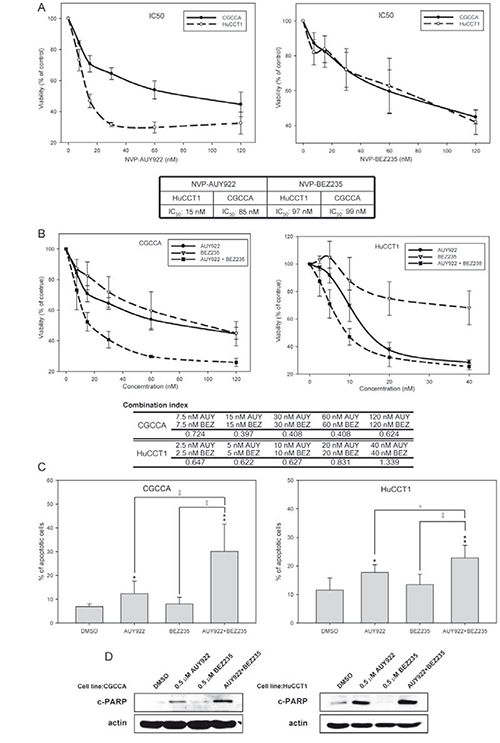
The combination of NVP-AUY922 and NVP-BEZ235 synergistically induced apoptosis in CCA cell lines **(A)** CGCCA and HuCCT1 cells were incubated with various concentrations (0, 8, 16, 32, 64, or 128 nM) of either NVP-AUY922 or NVP-BEZ235 for 72 h; **(B)** CGCCA and HuCCT1 cells were incubated with either NVP-AUY922 or NVP-BEZ235 or both NVP-AUY922 and NVP-BEZ235 at various concentrations for 72 h. The combination index (CI) < 1, CI = 1, or CI > 1 indicate synergism, an additive effect, or antagonism, respectively; **(C)** CGCCA and HuCCT1 cells were treated with 0.5 uM of either NVP-AUY922 or NVP-BEZ235 or a combination of these 2 for 72 h. The number of apoptotic cells measured using the TACS Annexin V-FITC apoptosis detection kit is represented as a percentage of the total events. **(D)** The immunoblots are analyses of cleaved poly (ADP-ribose) polymerase (PARP). β-Actin was used as the loading control.

The effects of NVP-AUY922 or NVP-BEZ235 mono-therapy and the combination on the growth of CCA cell lines were determined. NVP-AUY922 and NVP-BEZ235 exhibited strong antiproliferative effects in both HuCCT1 and CGCCA cells. In HuCCT1 and CGCCA cells, the IC_50_ values of NVP-AUY922 were 15 and 85 nM, respectively, and those of NVP-BEZ235 were 97 and 99 nM, respectively (Figure 2A). NVP-AUY922 (at 7.5, 15, 30, 60, and 120 nmol/L) at NVP-BEZ235 (at 7.5, 15, 30, 60, and 120 nmol/L) both alone and in combination induced dose-dependent cytotoxicity in the CGCCCA cells (Figure 2B). The combination of NVP-AUY922 and NVP-BEZ235 induced synergistic cytotoxicity. Similar results were seen in the HuCCT1 cell line (Figure 2B). Although NVP-BEZ235 itself did not trigger cell death in either cell line, both PI-staining and PARP cleavage analysis (Figure 2C) revealed that it synergistically activated apoptotic activity with NVP-AUY922.

### NVP-AUY922 and NVP-BEZ235 work together to block the PI3K/Akt/mTOR signaling pathway

**Figure 3:**
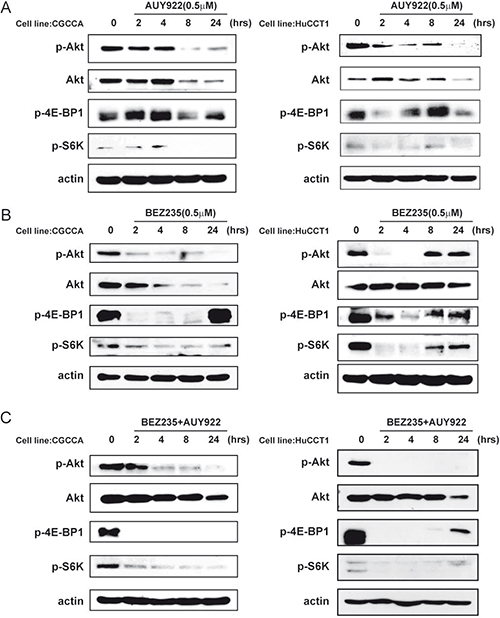
NVP-AUY922 and NVP-BEZ235 functioned together to block the PI3K/Akt/mTOR signaling pathway **(A)** Western blot analysis revealed the molecular signature of PI3K/mTOR inhibition induced by NVP-AUY922 in the CCA and HuCCT1 cell lines. Cell lysates from the CGCCA and HuCCT1 cell lines were treated with 0.5 uM NVP-AUY922 at various time points (0, 24, 48, 72 h); **(B)** Western blot analysis reveals the molecular signature induced by NVP-BEZ235 in CCA and HuCCT1 cell lines. Cell lysates from CGCCA and HuCCT1 cells were treated with 0.5 uM NVP-BEZ235 for various time points (0, 24, 48, and 72 h); **(C)** Western blot analysis revealed the molecular signature induced by NVP-BEZ235 and NVP-AUY922 treatment in CCA and HuCCT1 cell lines. Cell lysates from CGCCA and HuCCT1 cells were treated with 0.5 uM NVP-AUY922 and NVP-BEZ235 at various time points (0, 24, 48, and 72 h). β-Actin was used as the loading control.

To analyze the combinatorial effect of a PI3K/mTOR dual inhibitor and an HSP90 inhibitor, western blotting was performed to analyze the activation status of a series of proteins in the PI3K/Akt/mTOR pathway. NVP-BEZ235 efficiently inhibited both PI3K and mTOR activity within 2 h as indicated by the phosphorylation status of Akt, 4E-BP1, and S6K, respectively. However, this was abrogated in the 8–24-h time period (Figure 3B). On the other hand, NVP-AUY922 inhibited this pathway more slowly (Figure 3A). Interestingly, the kinetics of PI3K/Akt/mTOR signaling pathway inhibition by the combination treatment was similar to that of NVP-BEZ235 alone. Moreover, the combination markedly hindered abrogation of the effect of NVP-BEZ235 mono-therapy (Figure 3C). These results showed that NVP-BEZ235 and NVP-AUY922 work together to block the PI3K/Akt/mTOR signaling pathway.

### NVP-AUY922 induced ER stress and disrupted mitochondrial homeostasis

**Figure 4:**
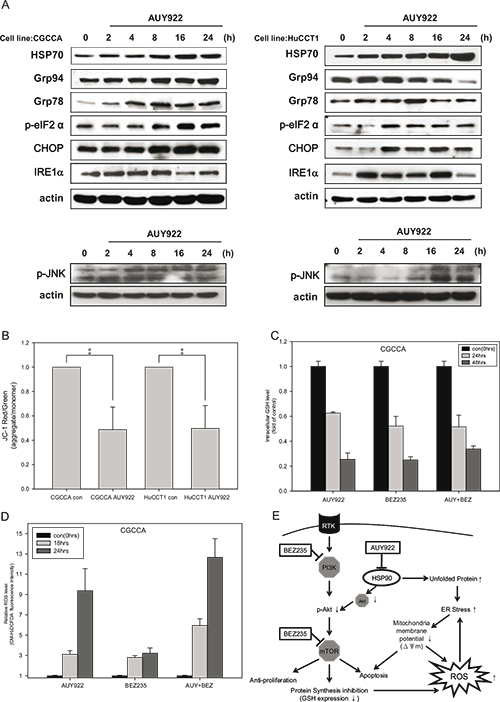
NVP-AUY922 induced ER stress and mitochondrial damage, which was fueled by oxidative stress when combined with NVP-BEZ235 **(A)** CCA cells were incubated with 0.5 uM NVP-AUY922 for 0, 2, 4, 8, 16, and 24 h. Whole cell lysates were subjected to western blot analysis for HSP70, Grp94, Grp78, p-eIF2α, CHOP, IRE1α, and phosphor-JNK. β-Actin was used as the loading control; **(B)** CCA cells were incubated with 0.5 uM NVP-AUY922 for 48 h. The red and green color ratio of JC-1 reflects the change in the mitochondrial membrane potential (ΔΨm); **(C)** Relative levels of reduced glutathione (GSH) in CGCCA cell line treated with 0.5 uM NVP-AUY922 and NVP-BEZ235 alone or combined for 0, 24, and 48 h. **(D)** Reactive oxidative species (ROS) levels induced by 0.5 uM NVP-AUY922 and NVP-BEZ235 alone or combined for 0, 18, and 24 h in CGCCA cell line. **(E)** The model shows that NVP-AUY922 induces ER stress, which leads to mitochondrial damage, and ultimately to apoptosis. When combined with NVP-BEZ235 treatment, this process is fueled by oxidative stress. NVP-BEZ235 and NVP-AUY922 cooperate to induce apoptosis by vertically affecting the PI3K/Akt/mTOR signaling pathway at multiple nodes.

We analyzed whether the HSP90 inhibitor NVP-AUY922 triggered ER stress in CCA cells. Indeed, NVP-AUY922 upregulated the expression of several chaperones such as Grp94, Grp78, IRE1α and phosphorylation of eIF2α (Figure 4A). These signaling signatures represent the unfolded protein response (UPR) [28] and indicate that CCA cells were struggling to survive under NVP-AUY922 treatment. However, CHOP and JNK activation indicated a shift to the ER stress-induced apoptosis response in CCA cells following NVP-AUY922 treatment [Figure 4A, 29–31]. These data are consistent with the observation that NVP-AUY922 alone triggered a collapse in mitochondrial membrane potential (Figure 4B) and induced CCA cell apoptosis (Figure 2C).

### NVP-AUY922 and NVP-BEZ235 promoted excessive oxidative stress by inducing ROS and simultaneously suppressing the G6PD/glutathione antioxidant pathway

NVP-AUY922 induced UPR and ER stress, which led to mitochondrial damage and cell apoptosis. This process might be exacerbated by oxidative stress when combined with an mTOR inhibitor [27]. Oxidative stress is caused by an imbalance between ROS production and ROS clearance. We investigated whether NVP-BEZ235 might enhance the effects of NVP-AUY922 by suppressing endogenous antioxidant levels. Interestingly, Both NVP-BEZ235 and NVP-AUY922 alone or combined decreased the reduced form of glutathione (GSH), one of the most important endogenous antioxidants, in CCA cells (Figure 4C and supplemental Figure 1A). We also analyzed whether simultaneously inhibiting HSP90 and the PI3K/AKT pathway could induce strong ROS production. Our result demonstrated that the combination of NVP-AUY922 and NVP-BEZ235 triggered strong ROS production in CCA cells (Figure 4D and supplemental Figure 1B).

### Evaluation of the antitumor effect of NVP-AUY922 and NVP-BEZ235 on CCA *in vivo*

**Figure 5:**
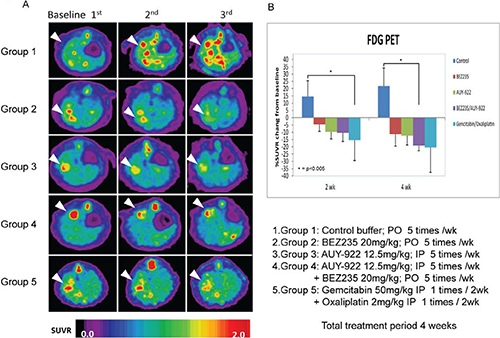
Detection of rat CCA by animal PET and changes in the tumor/liver SUV ratio **(A)** Coronal views of fused CT and PET scans of control and experimental rats revealed the CCA-expressing areas of the liver in which the ^18^F-FDG uptake was higher than baseline at 2–5 wk after the experiment (i.e., wk 20, 22, and 25). **(B)** Change in the tumor-to-liver (T/L) ratio of SUV in the control and experiment groups at 2–5 wk after the experiment (i.e., wk 22 and 25).

After inducing CCA with TAA, tumors in the control and treated groups were evaluated by transverse, sagittal, and coronal PET-computed tomography (CT) images. As shown in Figure 5A, each group had at least one FDG-avid tumor in the liver after 20 weeks of TAA treatment, as demonstrated by animal the PET-CT coronal images. In the experimental groups, rats were given BEZ235 alone (Group 2), AUY922 alone (Group 3), BEZ235/AUY922 (Group 4), or gemcitabine/oxaliplatin (Group 5). The change in the tumor-to-liver (T/L) ratio, as indicated by the SUV for each group, is shown in Figure 5B. The T/L ratio of SUV in the control group (Group 1) elevated steadily until the last scan (a 14.5% to 21.7% increase, from the second to the fifth week after the experiments; Figure 5B). The T/L ratio of SUV in each treatment group tended to decrease until the last scan; the T/L ratio of SUV decreased 2 weeks after the experiment, ranging from 3% to 15%. Gemcitabine with oxaliplatin induced a significant decrease in the T/L ratio of SUV after 2 weeks compared with the control group (control, 14.7% versus group 5, −15.5%, *p* < 0.05; Figure 5B). However, after 5 weeks of treatment, NVP-AUY922 and NVP-BEZ235 induced a significant decrease in the T/L ratio of SUV compared to the control group (control, 21.7% versus group 4, −18.3%; *p* < 0.05; Figure 5B).

## DISCUSSION

In this study, we demonstrated that both high HSP90 expression and loss of PTEN expression were independent prognostic factors in CCA. This implies that HSP90 and the PTEN-related PI3K/Akt/mTOR pathway are potential therapeutic targets in CCA. We then investigated the effect of a combination of an HSP90 inhibitor (NVP-AUY922) and a PI3K/mTOR dual inhibitor (NVP-BEZ235) on CCA. We found that they exerted significant synergistic proapoptotic and antiproliferative effects in CCA cell lines *in vitro*. This combination worked in concert to inhibit the PI3K/Akt/mTOR pathway and induce ROS accumulation; these effects may exaggerate the vicious cycle of ER stress. We also demonstrated the synergistic effect of the NVP-AUY922 and NVP-BEZ235 in a TAA-induced CCA animal model.

HSP90 is a ubiquitously expressed chaperone that is involved in the post-translational folding and activation of numerous client proteins implicated in oncogenesis [32]. The prognostic value of HSP90 expression in several types of cancer has been discussed, but to our knowledge, there is the first report of the clinical significance of HSP90 expression in CCA prognosis. Lower HSP90 protein expression was associated with longer overall survival (Table 3 and Figure 1). Congruently, HSP90 expression was significantly associated with tumor aggressiveness and poor prognosis in gastric and breast cancer [33, 34], which indicates HSP90 is a good drug target in CCA [11, 35].

Moreover, the PI3K/Akt/mTOR pathway is upregulated in CCA cells [22] and is one of the most important targets for CAA drug development [6, 7]. Activating mutations in *PIK3CA* are only found in 9% of intrahepatic CCA cases [22, 36], suggesting that additional mechanisms may positively regulate this pathway. For instance, *PTEN*, a well-characterized human tumor suppressor gene, is an antagonist of the PI3K/Akt/mTOR pathway. Loss of *PTEN* and activation of SMAD4 or KRAS activation may induce CCA development in murine models [37, 38], however, clinical implications of decreased PTEN protein expression in intrahepatic CCA has not yet been investigated. In this study, univariate analyses indicated that loss of PTEN expression correlated with a worse survival in patients with intrahepatic CCA (Table 3 and Figure 1). This result also supports our published data; PI3K/Akt/mTOR pathway plays a critical role in CCA [11]. Moreover, patients with high HSP90 protein expression and PTEN loss had the worst survival according to multivariate analyses (Table 4), which suggested that combining a PI3K inhibitor with an HSP90 inhibitor may represent an effective treatment for CCA.

The primary function of HSP90 in cancer was thought to be stabilization of client oncoproteins, suggesting that this protein might be a good drug target. However, recent HSP90 inhibitor clinical trials have shown that use of HSP90-targeted drugs might not be an optimally effective therapeutic strategy [9]. Combining HSP90 inhibitors with other targeted therapies may block the compensatory signaling mechanisms and impart a clinical benefit [39]. One strategy is vertically targeting the same pathway [40]. For example, the survival of patients with melanoma and the BRAF V600 mutation improved when treated with a combined BRAF and MEK inhibitor [41]. In our previous study, we identified Akt as a client protein of HSP90 [11]. NVP-BEZ235 is a novel dual PI3K/mTOR inhibitor and, when combined with NVP-AUY922, may vertically target the pathway at multiple nodes (Figure 4E). Indeed, we observed that combining NVP-AUY922 and NVP-BEZ235 enhanced and prolonged the inhibition of the PI3K/Akt/mTOR pathway (Figure 3C).

A broad spectrum of insults, including nutrient deprivation, can trigger ER stress, leading to activation of adaptive pathways to alleviate the insults, restore ER homeostasis, and ensure survival. However, under extensive stress, this same system will trigger a pro-apoptotic pathway [29, 42]. Most cancer cells have chronically elevated baseline ER stress levels, as indicated by increased expression of GRP78 [43]. When treated with drugs that specifically trigger ER stress, the key pro-apoptotic ER stress protein, CHOP, can induce cancer cell apoptosis [44]. HSP90 is critical for several cellular functions, including protein folding and assembly. Thus, an HSP90 inhibitor may increase unfolded protein accumulation (UPR), increase ER stress, and cause apoptosis [26]. In our study, we found that NVP-AUY922 induced ER stress and activated the UPR, as demonstrated by the upregulation of GRP78, peIF2a, GRP94, IRE1α and CHOP, within 4 h (Figure 4A) in CCA cells. The ER stress induced apoptosis was via both the JNK and caspase 4 pathways (Figure 4A and supplemental Figure 2).

ER stress can lead to ROS production, which occurs subsequent to the accumulation of unfolded proteins in the ER. Mitochondrial ROS is also generated in after ER stress induced by Ca^2+^ release and depolarization of the inner mitochondrial membrane (ΔΨm) [26, 45]. Oxidative stress, along with unresolved ER stress, contributes to cell death. Furthermore, the HSP90 inhibitor IPI-504 and the mTOR inhibitor rapamycin exert synergic antitumor effects in Ras-driven tumors by promoting ER stress and mitochondrial damage, via IPI-504 mediated ROS increase and a rapamycin-dependent suppression of GSH [27]. We here observed that NVP-AUY922 induced ER stress and decreased the mitochondrial membrane potential (ΔΨm) (Figure 4A and 4B). Both NVP-AUY922 and NVP-BEZ235 inhibited GSH synthesis (Figure 4C and supplemental Figure 1A). Finally, both NVP-AUY922 and NVP-BEZ235 induced ROS production (Figure 4D and supplemental Figure 1B) and promoted cell death.

To investigate the effectiveness of NVP-AUY922, NVP-BEZ235, their combination and gemcitabine and oxaliplatin for CCA treatment, we used the previously established TAA-induced rat CCA model [46]. This model recapitulates the histological progression of human CCA [46], indicating that it is a good platform to investigate new CCA treatment regimens. PET was then used to measure the tumor response to treatment [47]. Because we could not detect lesions < 2 mm by PET and the invasive CCA border was indistinguishable from the normal liver background, the T/L ratio of the SUV was used to represent tumor growth [47]. The T/L ratio of the SUV was significantly lower in rats treated with NVP-AUY922 and NVP-BEZ235 than in control rats by 5 weeks after treatment (Figure 5B), suggesting that tumor growth was suppressed in rats treated with NVP-AUY922 and NVP-BEZ235.

In conclusion, both HSP90 protein overexpression and lower PTEN expression were poor prognostic factors, which implied that HSP90 inhibitors and PI3K/mTOR inhibitors are potential therapeutic agents in CCA. NVP-AUY922 and NVP-BEZ235 had potent synergic antitumor activity (in the low nanomolar range) against CCA cells. Marked tumor regression was demonstrated in the TAA-induced CCA rat animal model upon treatment with a combination of NVP-AUY922 and NVP-BEZ235. These preclinical findings provide a rationale to conduct clinical trials with NVP-AUY922 and NVP-BEZ235 in CCA patients.

## METHODS

### Clinicopathological features of 78 patients with mass-forming CCA (MF-CCA)

From the archives of the Chang Gung Memorial Hospital, 78 patients with MF-CCA who had undergone hepatectomy between 1989 and 2006 were selected based on the availability of sufficient quantities of tumor cells. Intrahepatic CCA was defined as a carcinoma that had arisen from the second-order or more distal branches of the intrahepatic ducts. CCAs were further classified into 3 types based on the macroscopic appearance of the tumors: MF-CCA, periductal-infiltrating CCA, and intraductal papillary CCA. Curative resection was defined as a negative resection margin observed during histopathological examination. Surgical mortality was defined as death within a month of surgery. Laboratory tests were conducted the day before the surgery. Serum carbohydrate antigen 19-9 (CA 19-9) and carcinoembryonic antigen (CEA) levels were measured by radioimmunoassay. Tumors were preoperatively evaluated by abdominal ultrasonography (US), endoscopic retrograde cholangiopancreatography, percutaneous transhepatic cholangiography, computed tomography (CT), magnetic resonance cholangiopancreatography (MRCP), or hepatic arteriography, as appropriate. Tumor stage was defined according to the pathological tumor node metastasis (pTNM) classification proposed by the American Joint Committee on Cancer (AJCC), 6^**th**^ edition. Following either a positive section margin or local recurrence, adjuvant chemotherapy was systemically administered with a 5-flurouracil (5-FU)-based regimen. This clinical study was approved by the Institutional Review Board (IRB) of the Chang Gung Memorial Hospital (No. 99-2886B). All patients gave informed consent prior to inclusion in the immunostaining study.

### Immunohistochemical staining of HSP90 and PTEN in 78 MF-CCA

Hematoxylin and eosin (H&E)-stained slides from each case were reviewed. Specimens from MF-CCA patients who had undergone hepatectomy were fixed in formalin and embedded in paraffin. A 4-μm section was stained for HSP90 and PTEN. Primary antibodies against HSP90 (#3098-100, 1:200 dilution; BioVision, Mountain View, CA) and PTEN (#ab31392, 1:100 dilution; Abcam, Cambridge, UK) were added to the slides, and the slides were incubated overnight at 4°C. The slides were then washed in TBST 3 times for 5 min each before visualization with the DAKO LSAB2 System, Peroxidase (no K0675; DAKO A/S, Glostrup, Denmark). Control slides were incubated with the secondary antibody only. After washing in TBST 3 times for 5 min each, the slides were mounted. The slides were analyzed blindly under microscopy, and the expression in the cholangiolar epithelium was classified as low or high according to the following: negative (< 1% cytoplasmic staining), 1+ (1%–20% cytoplasmic staining), 2+ (21%–50% cytoplasmic staining), or 3+ (> 50% membranous staining). Negative and 1+ immunostaining were arbitrarily classified as low expression, while all others were classified as high expression.

### Follow-up study

The follow-up evaluation included a physical examination and blood chemistry tests at each visit. In addition, serum levels of CEA and CA 19-9 were measured, and the remnant liver was examined by US every 3 months. When a new lesion was detected by US or elevated levels of CEA/CA 19-9 were noted, abdominal CT or MRCP was performed for confirmation. Moreover, when patients complained of bone pain, bone scans were performed to detect metastasis. If any of the abovementioned procedures indicated recurrence, the patient was re-admitted for a more comprehensive assessment, including angiographic evaluation or magnetic resonance imaging (MRI). Treatment for recurrence included surgery, systemic chemotherapy, external beam radiotherapy, intraluminal radiotherapy, interventional radiological therapy, and conservative treatment.

### Cell lines

Two intrahepatic CCA cell lines, HuCCT1 and CGCCA, were obtained from the Japanese Collection of Research Bioresources (Osaka, Japan) and Chang Gung Memorial Hospital, respectively [46, 48]. HuCCT1 and CGCCA cells were routinely cultured in RPMI 1640 and Dulbecco's modified Eagle's medium (Gibco, Grand Island, NY), respectively, supplemented with 10% heat-inactivated fetal bovine serum, 100 μg/mL streptomycin, and 100 μg/mL penicillin, in a humidified atmosphere containing 5% CO_2_ at 37°C.

### Reagents

NVP-AUY922 and NVP-BEZ235 were provided by Novartis (Basel, Switzerland). Gemcitabine and oxaliplatin were purchased from TTY Biopharm (Taipei, Taiwan). For the *in vitro* experiments, 10 mM stock solutions of NVP-AUY922 and NVP-BEZ235 were prepared in 100% DMSO and stored at −20°C (NVP-AUY922) and 4°C (NVP-BEZ235). For administration, optimized NVP-AUY922 and NVP-BEZ235 salts with high solubility in aqueous solution were formulated in D5W. NVP-AUY922 was delivered by intraperitoneal (ip) injection in a volume of 6.25 mg/kg. NVP-BEZ235 was delivered orally (po) in a volume of 15 mg/kg. Gemcitabine (50 mg/kg) and oxaliplatin (2 mg/kg) were delivered by ip injection.

### Cell viability assay

The viability of the cells was determined using the sulforhodamine B (SRB) (Sigma-Aldrich, St. Louis, USA) assay, it's based on measuring cellular protein content of live cells. The CGCCA and HuCCT1 cells were seeded with 2 × 10^3^/well in 100 μl of culture medium into 96-well microplates, and allowed to adhere for 24 h. Next day, the cells were treated with various concentration of NVP-AUY922, NVP-BEZ235 or in combination for 72 h. After the incubation period, the medium were removed, and the cells were fixed with 10% (W/V) trichloroacetic acid (TCA) (Sigma-Aldrich, St. Louis, USA) for 30 min at 4°C. Then, cleared up TCA and washed the plates with water four times, allowed them to air-dry at room temperature. Next step, stained with SRB dissolved in 1.0% acetic acid for 30 min, after which, rinsed the plates repeatedly with 1.0% acetic acid to remove excess dye and air-dry. The protein-bound dye was dissolved in 10 mM Tris base solution, and determine optical density (OD) at 540 nm using a VersaMax microplate reader (Molecular Devices, Sunnyvale, USA).

### Combination index (CI) determination

The CI values of NVP-AUY922 and NVP-BEZ235 were evaluated using a Biosoft CalcuSyn software program (Ferguson, MO) based on the method reported by Chou and Talalay [49]. Cells were treated with various concentrations of drugs either alone or in combination for 72 h. Data from cell viability assays were expressed as the fraction of antiproliferative activity (cell viability loss) induced by an individual drug, alone or in combination, and CI values were generated for the 2 drugs. CI < 1, CI = 1, and CI > 1 indicated synergism, an additive effect, or antagonism, respectively.

### Measurement of reactive oxidative species (ROS)

ROS generation was quantified using an oxidant-sensitive fluorescent probe (CM-H2DCFDA; Invitrogen, Carlsbad, CA) as a substrate for measuring intracellular oxidant production [50]. Cells were seeded into 6-well plates (1 × 10^**5**^ cells/well), incubated for 24 h, and then treated with NVP-AUY922 or NVP-BEZ235 for 18 or 24h. Next, these cells were incubated with CM-H2DCFDA (20 μM final concentration) for 30 min at 37°C, harvested, and washed twice with PBS; then, fluorescence was quantified by flow cytometry using a FACSCalibur (Becton Dickinson, Franklin Lakes, NJ). ROS production was expressed as the mean fluorescence intensity. Experiments were performed in triplicate, and data are expressed as mean ± standard deviation.

### Assay to detect apoptotic cell death

Apoptosis was measured using the TACS Annexin V-FITC apoptosis detection kit (Trevigen) according to the manufacturer's instructions. After a 24-h incubation, cells were treated with DMSO, NVP-AUY922, or NVP-BEZ235 for 48 h. Cells were collected and stained with Annexin V and propidium iodide (PI), and then analyzed using a FACSCalibur. The data were analyzed using CellQuest software (BD). Experiments were performed in triplicate, and data are expressed as mean ± standard deviation.

### Measurement of mitochondrial membrane potential (ΔΨm)

Variation in ΔΨm was investigated using 5,5',6,6'-tetrachloro-1,1',3,3'- tetraethyl-benzimidazol-carbocyanine iodide (JC-1; BD Biosciences, San Diego, CA) according to the manufacturer's instructions [51]. Cells were seeded into 6-well plates at a density of 1 × 10^**5**^ cells/well, incubated overnight, and then treated with DMSO or 0.5 uM NVP-AUY922 or NVP-BEZ235 or both. After treatment, the cells were collected and incubated with JC-1 for 15 min at 37°C. After washing twice with PBS, the cells were suspended in a total volume of 400 mL and analyzed using a FACSCalibur.

### Measurement of glutathione content

Intracellular reduced glutathione (GSH) content was determined using the GSH-Glo™ Glutathione Assay kit (Promega, Madison, WI) [52]. The kit is a luminescence-based assay for the detection and quantification of GSH. Briefly, cells were seeded into 96-well white plates (SPL Life Sciences, Korea) at a concentration of 3,000 (HuCCT1) or 1,800 (CGCCA) cells/well in 100 μL of culture medium. At 24 h post-seeding, the cells were treated with 0.5 uM NVP-AUY922 or NVP-BEZ235 or both for 24 or 48 h, and then processed according to the manufacturer's instructions. The luminescence values were measured using the Infinite M1000 microplate reader (TECAN, Australia).

### Western blotting

Whole cell lysates of CCA cell lines were obtained using Pierce RIPA buffer (Thermo Scientific, Rockford, IL). Protein samples were separated on 8%–12% gradient sodium dodecyl sulfate- polyacrylamide gels (SDS-PAGE) and transferred to Immobilon-P (Millipore, Bedford, MA) membranes. Antigen-antibody complexes were detected using the ECL blotting analysis system (Millipore). Primary antibodies against the following targets were used: AKT (9272; Cell Signaling, Danvers, MA), p-AKT (9271; Cell Signaling), cleaved poly (ADP-ribose) polymerase (c-PARP, 9541S; Cell Signaling), p-4E-BP1 (9459; Cell Signaling), p-p70S6K (9205; Cell Signaling), β-actin (Abcam ab6276; Abnova Corporation, Taipei), HSP70 (4872; Cell Signaling), Grp78 (BiP, 3183; Cell Signaling), Grp94 (2104; Cell Signaling), p-eIF2α (3597; Cell Signaling), Caspase 4 (4450; Cell Signaling), IRE1α (3294; Cell Signaling), phosphor-JNK (9255; Cell Signaling), and CCAAT/-enhancer-binding protein homologous protein (CHOP, GADD153, sc-575; Santa Cruz Biotechnology, Santa Cruz, CA).

### Animal studies

All animal studies were approved by the experimental animal ethics committee of the Chang Gung Memorial Hospital, and were conducted in accordance with the US National Institute of Health (NIH) guidelines for the care and use of laboratory animals (Publication no. 85-23, revised 1996). Thirty adult male Sprague-Dawley (SD) rats (310 ± 14 g) were equally divided into 5 groups: the control (Group 1), BEZ235 (Group 2), AUY922 (Group 3), BEZ235/AUY922 (Group 4), and gemcitabine/oxaliplatin groups (Group 5). The rats were housed in an animal room under a 12-h light-dark cycle (light from 08:00 AM to 08:00 PM) at an ambient temperature of 22°C. Food and water were provided *ad libitum*. The rats were administered 300 mg thioacetamide (TAA)/L in drinking water daily for up to 25 weeks. Mice in the BEZ235, AUY922, and BEZ235/AUY922 groups were administered NVP-BEZ235 (15 mg/kg, po), NVP-AUY922 (6.25 mg/kg, ip), or a combination of these 2 agents, respectively, once daily 5 d per week over a 4-week period (specifically, the 21^**st**^ week to the 24^**th**^ week of the experimental period). The gemcitabine/oxaliplatin group received gemcitabine (50 mg/kg, ip) and oxaliplatin (2 mg/kg, ip) once every 2 weeks over a 4-week period. The control group rats received ip injections of buffer, according to the same schedule.

### Positron emission tomography

To evaluate changes in glycolysis in live rats with liver tumors, we conducted 2-deoxy-2-[F-18] fluoro-d-glucose (FDG)-positron emission tomography (PET) studies at the molecular imaging center of Chang Gung Memorial Hospital. Thirty rats were treated with thioacetamide and subjected to serial PET scanning on weeks 20, 22, and 25 using the Inveon™ system (Siemens Medical Solutions USA Inc., Knoxville, TN). Equal numbers of animals were assigned to the control and treatment groups according to baseline PET findings. In other words, the control and treatment groups possessed similar PET-positive rates. The details regarding radioligand preparation, scanning protocols, and optimal scanning time determination have been described previously by our group [47]. Briefly, animals were fasted overnight prior to the scan. At 90 min post-^**18**^F-FDG injection (intravenous), 30-min static scans were obtained for all the animals. All imaging studies were performed using a temperature- (37°C) and anesthesia gas- (2% isoflurane in 100% oxygen) controlled imaging bed (Minerve System). PET images were reconstructed using the 2D ordered subset expectation-maximization method (4 iterations and 16 subsets) without attenuation and scatter corrections. All imaging data were processed using the PMOD image analysis workstation (PMOD Technologies Ltd., Zurich, Switzerland). The largest liver tumor was identified by careful investigation of 3 image sets for each rat. ^18^F-FDG uptake into the largest liver tumor and apparently normal liver tissue was quantified by calculating the standardized uptake value (SUV) according to the following formula:

$$\text{SUV}=\frac{\text{Decay corrected tissue acitivity (Bq/ml) }}{\text{Injected dose (Bq)/Body weight(g)}}$$

These values were calculated according to the recommendations of the European Organization for Research and Treatment of Cancer [43]. The tumor regions of interest (ROIs) were determined using transverse images of the selected tumors and measuring the largest diameter. Normal liver ROIs were also determined using the same transverse images. The mean SUV (SUV_mean_) of the normal liver and tumor tissue was determined, and the tumor-to-liver radioactivity ratio was calculated for comparison.

### Statistical analysis

All data are presented as mean ± standard deviation (SD). Differences between experimental animals and controls were calculated using the Mann-Whitney *U* test or the Kruskal-Wallis test. Overall survival was calculated using the Kaplan-Meier method. Eighteen clinicopathological variables were selected for difference analysis using the log-rank test (univariate). The Cox proportional hazards model was employed for multivariate regression analysis. SPSS statistical software for Windows was used for statistical analysis (SPSS version 13.0; Chicago, IL). *P* values ≤ 0.05 were considered statistically significant.

## SUPPLEMENTARY FIGURES AND TABLES


