# Supplementary material for: Antitumor activity of the combination of an HSP90 inhibitor and a PI3K/mTOR dual inhibitor against cholangiocarcinoma
Source: Oncotarget. 2014 Mar 26;5(9):2372–89. doi: 10.18632/oncotarget.1706 (PMC4058012; doi:10.18632/oncotarget.1706)
Supplement: Supplementary file 1 [file oncotarget-05-2372-s001.pdf]

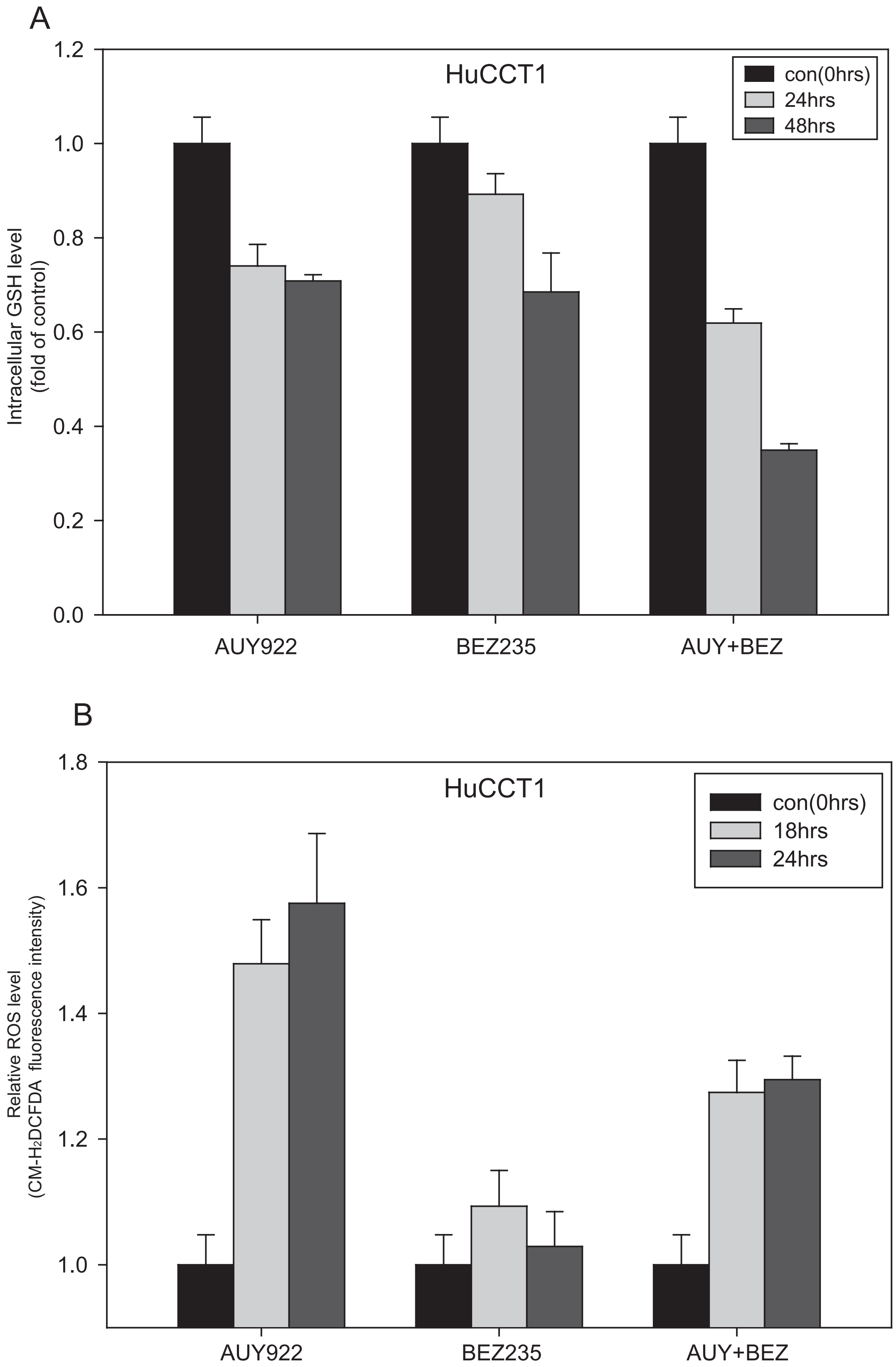

Supplementary Figure S1: NVP-AUY922 and BEZ235 induced Reactive oxidative species (ROS) and reduced glutathione(GSH) in HuCCT1 cell line. **(A)** Relative levels of reduced glutathione (GSH) in HuCCT1 cell line treated with 0.5 uM NVP-AUY922 and NVP-BEZ235 alone or combined for 0, 24, and 48 h. **(B)** Reactive oxidative species (ROS) levels induced by 0.5 uM NVP-AUY922 and NVP-BEZ235 alone or combined for 0, 18, and 24h in HuCCT1 cell line.1

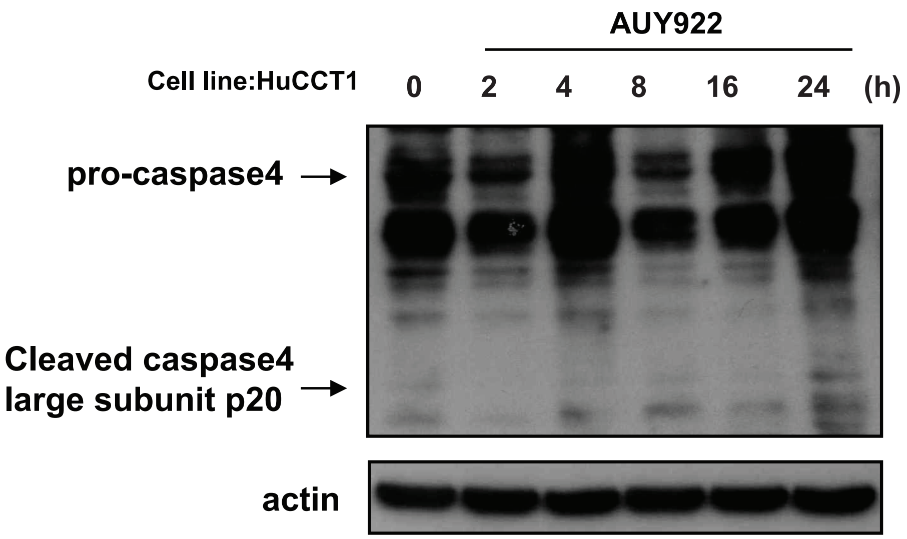

Supplementary Figure S2: NVP-AUY922 induced ER stress and apoptosis through Caspase 4 activation. HuCCT1 cells were incubated with 0.5 uM NVP-AUY922 for 0, 2, 4, 8, 16, and 24 h. Whole cell lysates were subjected to western blot analysis for caspase 4. β-Actin was used as the loading control.
